# Bridging dream and wake states: a new tool for consciousness research

**DOI:** 10.1093/nc/niag038

**Published:** 2026-07-15

**Authors:** Zeynep Temel Başer, Gülsüm Uzer, Hansa Şentürk, Ali Yalçınkaya, Tuba İkbal Yayla, Merve Dikmen, Halil Aziz Velioğlu, Erol Yıldırım, Lütfü Hanoğlu

**Affiliations:** Brain and Cognition Research Center (BEYKOG), Istanbul Medipol University, Istanbul, Türkiye; Department of Psychology, Istanbul Medipol University, Istanbul, Türkiye; Brain and Cognition Research Center (BEYKOG), Istanbul Medipol University, Istanbul, Türkiye; Brain and Cognition Research Center (BEYKOG), Istanbul Medipol University, Istanbul, Türkiye; Brain and Cognition Research Center (BEYKOG), Istanbul Medipol University, Istanbul, Türkiye; CogRes Lab, Fatih Sultan Mehmet Vakif University, Istanbul, Türkiye; Brain and Cognition Research Center (BEYKOG), Istanbul Medipol University, Istanbul, Türkiye; Brain and Cognition Research Center (BEYKOG), Istanbul Medipol University, Istanbul, Türkiye; Brain and Cognition Research Center (BEYKOG), Istanbul Medipol University, Istanbul, Türkiye; Department of Psychology, Istanbul Medipol University, Istanbul, Türkiye; Brain and Cognition Research Center (BEYKOG), Istanbul Medipol University, Istanbul, Türkiye; Department of Neurology, School of Medicine, Istanbul Medipol University, Istanbul, Türkiye

**Keywords:** dream consciousness, phenomenology of consciousness, REM sleep, self-report instrument, waking consciousness

## Abstract

In this study, the Consciousness States Comparison Scale: REM Version (CSCS-REM) was developed as a self-report instrument to assess dream consciousness during REM sleep in comparison with waking consciousness. Existing instruments capture only limited aspects of phenomenology and provide few opportunities for systematic cross-state evaluation. By anchoring waking consciousness as a reference point, the CSCS-REM quantifies relative differences in phenomenological experience and serves as a tool for empirically testing the predictions of the continuity and discontinuity hypotheses in consciousness research. The item pool was derived from a systematic review, expert evaluations, and preliminary studies, with content validity ensured through expert panels. After pilot testing, the 63-item draft was administered online, and data from 432 participants were analyzed using exploratory factor analysis. The final version consisted of 35 items across eight subscales: Metacognitive Awareness, Cognitive Fluency, Memory, Temporal Awareness, Spatial Awareness, Emotional Awareness, Perceptual and Bodily Senses, and Interoceptive and Chemical Senses. This structure accounted for 56.04% of the variance, with Cronbach’s α ranging from .736 to .915 and McDonald’s ω from .639 to .901. Confirmatory factor analysis in an independent sample (*n* = 211) demonstrated excellent fit indices (Comparative Fit Index = .986, Tucker-Lewis Index = .984, Root Mean Square Error of Approximation = .059). Subscales of the CSCS-REM showed small to moderate, yet significant, correlations with the Bodily Self-Consciousness in Dreams Questionnaire and the Dream Reflective Awareness Questionnaire, supporting convergent validity. In conclusion, while subject to some limitations, the CSCS-REM is a psychometrically reliable and valid instrument that enables systematic comparison of REM dream consciousness and waking consciousness across shared phenomenological dimensions.

Highlights

- We introduce a self-report instrument that compares REM dream consciousness with waking consciousness across shared emotional, cognitive, sensory, and bodily domains.
- Psychometric analyses identify a stable eight-factor, 35-item structure with acceptable-to-high internal reliability and evidence of construct validity.
- The scale captures both continuity and divergence between REM dreaming and waking experience, including bodily self-awareness and interoceptive experience.
- This instrument addresses a methodological gap by providing a standardized framework for empirical research on consciousness across sleep–wake states.

## Introduction

Consciousness has long been a central topic of inquiry in philosophy, psychology, and neuroscience. Its operationalization as a measurable construct, however, became possible only in the mid-20th century with advances in cognitive science, phenomenological methods, and objective measurement techniques (Aserinsky and Kleitman 1953; Seth 2018; Lambert 2022). Accumulating evidence indicates that consciousness extends beyond the waking state, encompassing alternative modes, such as dream consciousness during rapid eye movement (REM) sleep (Sanders et al. 2012; Koch et al. 2016; Tononi et al. 2024).

REM-dream and waking consciousness overlap at neurophysiological and phenomenological levels alike. At the same time, they diverge in several important respects. The relationship between these two states has been conceptualized in the literature along a continuity-discontinuity dimension: the continuity hypothesis holds that dreaming and waking share fundamental cognitive and affective processes, whereas the discontinuity hypothesis emphasizes qualitative differences arising from altered neurophysiological conditions during sleep (Domhoff 2017; Horton 2017). From a continuity perspective, these states share a range of cognitive and perceptual features (Kahan et al. 1997; Domhoff 2010; Wamsley and Stickgold 2010; Kahan and LaBerge 2011; Fox et al. 2013; Wamsley 2014; Malinowski and Horton 2021). Participants commonly report reflective self-awareness, intense affective engagement, attentional focus, and internal evaluation in either condition (Schredl and Reinhard 2010; Lee and Kuiken 2015). Emotionally salient daily events are often selectively incorporated into dream content, retaining their subjective tone and meaning associations (Hall and Nordby 1972; Schredl and Hofmann 2003). This body of evidence suggests that REM dream consciousness is not reducible to random associations or fragmented thought, but constitutes an organized ‘stream of consciousness’ comparable to waking mentation.

At the same time, the two states show clear structural and functional divergences. In REM dreaming, logical coherence and orientation are weakened; reality monitoring and narrative organization are also altered (Purcell et al. 1986; Hobson et al. 2000; Kahn et al. 2000; Occhionero and Cicogna 2016). Indeed, analytic cognitive activities such as reading, writing, and arithmetic are strikingly rare in dream reports (Hartmann 2000). Metacognitive activity is reduced, leaving reflective thought and the capacity to monitor one's own cognitive processes at a limited level (Cicogna and Bosinelli 2001; Occhionero and Cicogna 2016). The affective tone can also shift: dreams may become hyperemotional (Schredl and Doll 1998; Schredl and Reinhard 2010; Schredl 2013), and memory functions are often impaired or even blocked (Cohen 1971; Butler and Watson 1985; Blagrove and Pace-Schott 2010; Eichenlaub et al. 2014; Horton 2017; Fazekas et al. 2019; Windt 2020; Nemeth 2023). Examining these contrasts is central to consciousness research, as it enables testing of continuity and discontinuity claims and identification of cognitive and affective processes specific to each state. Several complementary theoretical frameworks have been proposed to account for the similarities and differences between states of consciousness, each addressing different aspects of dream generation, function, or phenomenological structure.

Neurocognitive theory conceptualizes dream production as a shift in neural networks, active during wakefulness, into an ‘internal simulation’ mode that operates independently of external sensory input. This approach is consistent with findings on continuity in content and affect between dreaming and waking, while also interpreting the narrative discontinuities and logical leaps of dreams as natural consequences of this mode shift (Domhoff 2001, 2003). The Activation-Input-Modulation model posits that both wakefulness and REM sleep are characterized by high levels of cortical activation; however, during REM, sensory input becomes ‘gated inward,’ and the balance of neuromodulation shifts in favor of the cholinergic system and against the monoaminergic system (Hobson 1988, 1997; Hobson et al. 2000). Extending this framework, the proto-consciousness theory conceptualizes REM sleep as a biological ‘preparation and rehearsal’ stage that supports the development, maintenance, and adaptive functions of waking consciousness; this process is particularly associated with the reorganization and optimization of cognitive functions such as sensorimotor integration during sleep (Hobson 2009). Within the broad predictive processing framework, one prominent model proposes that the brain operates during sleep through the same inference-based architecture as in wakefulness. However, during REM sleep, reduced sensory precision allows prior-based predictions to dominate information processing. This account suggests that the internally generated simulations of REM maintain continuity with waking architecture in terms of computational principles, while diminished sensory reliability and altered neuromodulatory conditions give rise to phenomenological divergence (Carhart-Harris and Friston 2010; Hobson and Friston 2012). Whereas the preceding models focus on the mechanisms of dream generation, other frameworks address the adaptive functions of dreaming. Threat Simulation Theory (TST) argues that dreams selectively rehearse threatening scenarios, thereby ‘training’ threat perception and avoidance mechanisms (Revonsuo 2000; Valli et al. 2005). This view is consistent with the high emotional arousal frequently observed during REM sleep. Whereas TST emphasizes threat rehearsal, Emotion Regulation/Fear Extinction accounts propose that emotional memory elements are reorganized during REM and re-associated with less negative contexts, thereby supporting both affective attenuation and reappraisal processes (Levin and Nielsen 2007). The Virtual Reality Model of Dreaming interprets dreams as a full-scale ‘internal virtual reality’ generated by the brain (Revonsuo 1995; Hobson et al. 2014). Consistent with the predictive processing account described above, this model holds that core cognitive processes are preserved during REM sleep; however, the closed-loop sensory environment means that the simulation is predominantly driven by internally generated data, giving rise to phenomenological differences. It should be noted, however, that the dreaming brain is not entirely disconnected from the sensory environment; evidence indicates that external stimuli can be partially incorporated into dream content (Solomonova and Carr 2019). The Multidimensional Phenomenology of Consciousness approach (Pekala 1991; Vaitl et al. 2005) emphasizes that consciousness is not a one-dimensional phenomenon, but consists of multiple components. Vaitl et al. (2005) characterize altered states along four dimensions: activation, awareness span, self-awareness, and sensory dynamics. Pekala (1991) proposes a broader framework encompassing 12 dimensions, including imagery, affect, self-awareness, volitional control, rationality, and memory. Together, these perspectives make it theoretically possible to measure and compare waking and dream consciousness along the same structural axes, while also providing a foundation for the development of phenomenological scales.

Testing these theoretical accounts and comparing the phenomenological structure of REM dream consciousness with waking consciousness requires self-report instruments. Consciousness cannot be fully captured by neurophysiological or behavioral indicators alone, because it involves qualia, the subjective dimension of experience that is inaccessible to direct third-person observation (Nagel 1974). Without tools that allow individuals to articulate their inner experiences in a systematic and reliable way, comparative analyses of consciousness remain methodologically incomplete. In large-scale studies, qualitative methods have limited applicability. Standardized self-report measures with established psychometric reliability and validity make it possible to capture phenomenological content quantitatively, ensuring both replicability and comparability across studies.

Several self-report instruments have been developed to assess dream consciousness (*e.g.* Phenomenology of Consciousness Inventory [PCI], Pekala 1991; Lucidity and Consciousness in Dreams [LuCiD], Voss et al. 2013; Subjective Experiences Rating Scale [SERS], Kahan and Claudatos 2016; Dreamland Questionnaire [DL-Q], Holzinger et al. 2020; Bodily Self-Consciousness in Dreams Questionnaire [BSD-Q], Erdeniz et al. 2023). These instruments capture important aspects of dream phenomenology. Among them, the PCI offers the broadest framework, assessing 12 dimensions of consciousness applicable across different states (Pekala 1991). However, two methodological gaps persist in the field. First, existing instruments either evaluate each state of consciousness independently (PCI), differentiate between dream subtypes (LuCiD), rate dream features in absolute terms (SERS, BSD-Q), or function primarily as structured dream diaries assessing content categories (DL-Q), but none embeds the comparison between dreaming and waking into the response format at the item level. This makes systematic within-subject comparison of the two states dependent on separate administrations and external comparison rather than on the measurement structure itself. Second, phenomenologically critical dimensions remain underrepresented. Spatial awareness and interoception are absent from all instruments reviewed. Temporal perception is addressed only minimally in the PCI. Metacognitive processes are limited to lucidity-specific items in the LuCiD or assessed at the level of general self-awareness in the PCI. The BSD-Q focuses exclusively on bodily sensory modalities, while the SERS does not explicitly assess metacognitive processes. These methodological gaps necessitated the development of a new instrument capable of assessing waking and REM dream consciousness comparatively, across the same phenomenological dimensions.

The aim of the present study was to create a psychometrically reliable and valid self-report scale that systematically and comparatively evaluates REM dream consciousness and waking consciousness within a shared framework encompassing cognitive, affective, perceptual, and sensory dimensions.

The scale was developed on the basis of specific theoretical assumptions. First, the phenomenological dimensions of conscious contents (*e.g.* sensory, perceptual, cognitive, affective, and metacognitive processes) are considered separable and measurable subcomponents. Second, REM dream consciousness and waking consciousness are assumed to be comparable, such that conscious experience can be evaluated in terms of relative deviations from wakefulness. It should also be noted that, as the scale relies on retrospective self-report, an inevitable gap between the measured experience and the actual phenomenological content is recognized as an inherent methodological constraint.

## Methods and materials

### Scale construction

In developing the CSCS-REM, the primary methodological concern was content validity (Skóra et al. 2021). A critical issue was whether the scale assessed the phenomenological difference between REM dream consciousness and waking consciousness, or merely reflected the intensity of recallable dream contents. To address this issue, the theoretical framework of the scale was grounded in findings from a systematic review. The review identified two major theoretical approaches: (i) models that conceptualize dream consciousness as a phenomenologically and neurocognitively distinct state, separate from waking consciousness (e.g. Rechtschaffen 1978; Hobson et al. 2000; Hobson 2009) and (ii) continuity models that view dream consciousness as an extension of waking consciousness (e.g. Foulkes 1990; Purcell et al. 1993; Domhoff 2003; Kahan and LaBerge 2011; Kahan and Sullivan 2012). The review also examined existing instruments related to REM dream consciousness, comparing their validity and reliability metrics, and found no standardized tool designed to directly contrast REM dream consciousness with waking consciousness. Consequently, the systematic review served as a foundation for constructing the item pool and defining the conceptual dimensions of the CSCS-REM.

To refine the conceptual domain of the construct, two complementary sources were consulted. First, 12 participants (7 female, 5 male; M = 23.67, SD = 4.29) listed differences between dream and waking consciousness based on dreams recalled within the past month. Five themes emerged from their reports: disruptions in logical coherence (*n* = 11), alterations in temporal orientation (*n* = 8), increased emotional intensity (*n* = 7; negative affect *n* = 5), changes in sensory/physical experience (*n* = 5), and impairments in spatial orientation (*n* = 2). Second, three clinicians working on dreams in their clinical practice (one psychoanalyst, one neurologist, and one psychiatrist) were asked which experiential domains they considered essential for a scale comparing REM dream and waking consciousness. This step was added to support the ecological validity of the instrument, since a purely theory-driven item pool could omit domains salient in clinical practice. Guided by these findings and the theoretical framework, the target construct of the scale was defined across the following subdomains: self, embodiment, volition/intentionality, awareness, sensation/perception, attention, memory, reasoning/planning, event coherence and causality, metacognition, motor/behavioral control, orientation (time/space), and emotion/affect. Following established recommendations in scale development (DeVellis 2003), an item pool approximately three times larger than the planned final scale was constructed. In the first step, 56 items were generated from prior theoretical and empirical studies (*e.g.*  Pekala 1991; Siclari et al. 2013; Genç et al. 2016). Then, 23 additional items were adapted from validated measures of REM dream phenomenology (*e.g.*  Kahan and Sullivan 2012; Voss et al. 2013). In total, a pool of 79 items was obtained.

Content and face validity were evaluated by an independent expert panel consisting of one neurologist, three psychiatrists, and three psychologists. Each item was rated as “Essential,” ‘Useful but requiring revision,’ or “Unnecessary.” The item-level content validity index (I-CVI) was calculated as the ratio of experts rating an item as “Essential” to the total number of experts, with a threshold of .78 adopted for item retention (Polit and Beck 2006). At the scale level, the Scale-level Content Validity Index using the averaging method (S-CVI/Ave ≥ .90) was adopted as evidence of satisfactory content validity (Polit et al. 2007). In addition, Kendall's coefficient of concordance revealed a weak-to-moderate but significant level of agreement among experts (W = .391, χ^2^(78) = 213.44, *P* < .001). Although statistical significance indicates that agreement exceeded chance levels, the magnitude of concordance suggests that expert opinions converged on some items more than others. Following this evaluation process, 16 items were excluded, and the final draft comprised 63 items.

To ensure linguistic clarity and accuracy, the draft scale was reviewed by two experts in Turkish Language and Literature affiliated with the university. The Turkish Linguistic Accuracy Form, which rates each item as ‘Accurate,’ ‘Needs revision,’ or ‘Inaccurate,’ was used for evaluation. Based on expert feedback, the necessary linguistic revisions were made, and the text was finalized in terms of grammar and expression. The scoring and response format was defined in consultation with a psychometrics expert who was not involved in the content validity process. Accordingly, a six-point Likert-type scale was adopted, with items scored as 0 = No response, 1 = Very low, 2 = Low, 3 = Same level, 4 = High, and 5 = Very high. The rationale of the CSCS-REM scoring system was to minimize terminological debates surrounding the concept of ‘levels of consciousness.’ Each experiential dimension was evaluated on a 0–5 scale; however, these scores reflected the degree of similarity or difference to waking consciousness rather than indicating higher or lower levels of consciousness. In this framework, waking consciousness functioned as an anchor, and dream experiences were positioned relative to this reference point. A score of 3 indicated equivalence to waking intensity, values below 3 represented a lower degree, and values above 3 represented a higher degree of difference. The inclusion of the ‘No response’ option was intended to reduce systematic errors arising from forced responses and to improve data quality. This option also accounted for cases where the content being evaluated was absent from participants’ dreams or could not be reported due to limitations in recall.

The finalized draft was subjected to a two-stage pilot testing process; no psychometric analyses were performed at this stage. The two stages served different purposes: the first pilot was conducted face-to-face to closely observe how participants understood the items, while the second pilot was conducted online to verify that the administration worked smoothly in the digital format used for the main data collection. The first pilot involved 16 participants (12 women, 75%; 4 men, 25%; M_age_ = 26.13, SD = 5.76), half with high school and half with a university degree or higher. The second pilot was conducted online with 11 high-school graduates (10 women, 90.9%; 1 man, 9.1%; M_age_ = 21.36, SD = 0.88). At both stages, after completing the scale in writing, participants provided written feedback on the clarity, order, and format of the items, the presence of any potentially disturbing expressions, and the comprehensibility of the instructions and scoring system. In the first pilot, the researcher additionally went through the items one by one in a semi-structured interview and asked each participant what they understood from each item; responses were recorded in writing. Items for which any negative feedback was received were revised at the wording level. After confirming that participants encountered no difficulty in understanding or completing the scale, data collection proceeded with the final 63-item version.

After the final factor structure was established through EFA and CFA (see Results), an additional preliminary feasibility test was conducted to evaluate whether the CSCS-REM could be administered in a laboratory setting. In the main study, data were collected in participants' home environments for a single dream on a single occasion. However, in laboratory-based sleep studies, the scale may need to be administered repeatedly across multiple REM periods throughout the night. This introduces additional requirements: participants must be able to comprehend the items despite sleep inertia, complete the scale within a reasonable time, and return to sleep afterward. As a preliminary step toward addressing these requirements, four participants (two female, two male; M_age_ = 22.25, SD = 2.06) underwent full-night polysomnography recordings. REM sleep was scored according to the American Academy of Sleep Medicine guidelines (Troester et al. 2023), based on low-amplitude, mixed-frequency EEG activity, low chin EMG tone, and rapid eye movements. Participants were awakened from REM sleep on a total of 13 occasions and the CSCS-REM was administered by a trained researcher. The average completion time was 10 minutes. Non-zero response rates exceeded 80% in 12 of the 13 awakenings, participants completed the items without comprehension difficulties, and all were able to return to sleep following each awakening. Across the 13 awakenings, mean subscale scores ranged from 1.64 (Interoceptive and Chemical Senses) to 2.97 (Emotional Awareness), with an overall mean of 2.53 (SD = 0.38).

### Participants

Two independent samples were recruited. The first sample was used for exploratory factor analysis (EFA) and the second for confirmatory factor analysis (CFA). Recruitment announcements were distributed through university email lists reaching both undergraduate/graduate students and academic/administrative staff, with detailed instructions on how to complete the online form; data were collected online in Türkiye. In both samples, participants were excluded if they reported not recalling their dreams, had neurological or psychiatric disorders or sleep-related problems, were using neurological/psychiatric or sleep medication, or failed the attention-check item. For the EFA sample, 1014 individuals were initially reached; after exclusions, the final sample consisted of 432 participants aged 18–65 years (M = 24.89, SD = 7.32, Mdn = 25.35). For the CFA sample, 309 individuals were initially reached; after exclusions, the final sample consisted of 211 participants aged 18–47 years (M = 22.64, SD = 4.91, Mdn = 21.00). Demographic, sleep, and dream-related characteristics of both samples are presented in Table 1.

**Table 1:** Demographic, sleep, and dream-related characteristics of the EFA and CFA samples

Variable	EFA (*n* = 432)		CFA (*n* = 211)	
	*n*	%	*n*	%
**Gender**				
Female	375	86.8	184	87.2
Male	57	13.2	27	12.8
**Education level**				
Secondary school	1	0.2	—	—
High school	30	6.9	11	5.2
Undergraduate	343	79.4	192	91.0
Master’s degree	43	10.0	7	3.3
Doctorate	15	3.5	1	0.5
**Average sleep duration (past year)**				
0–3 h	3	0.7	—	—
3–5 h	15	3.5	9	4.3
5–7 h	164	38.0	73	34.6
7–9 h	220	50.9	110	52.1
> 9 h	30	6.9	19	9.0
**Frequency of sharing dreams with others**				
Never	15	3.5	7	3.3
Less than once/year	9	2.1	2	0.9
About once/year	15	3.5	4	1.9
2–4 times/year	62	14.4	31	14.7
About once/month	86	19.9	47	22.3
2–3 times/month	91	21.1	49	23.2
Once/week	62	14.4	30	14.2
Several times/week	92	21.3	41	19.4
**Dream recording frequency**				
Never	263	60.9	138	65.4
Less than once/year	32	7.4	14	6.6
About once/year	20	4.6	9	4.3
2–4 times/year	27	6.3	17	8.1
About once/month	22	5.1	11	5.2
2–3 times/month	28	6.5	10	4.7
Once/week	15	3.5	6	2.8
Several times/week	25	5.8	6	2.8
**Age (years)**	*M* = 24.89, *SD* = 7.32		*M* = 22.64, *SD* = 4.91	

*Note.* Sleep duration, frequency of sharing dreams with others (*i.e.* verbally telling one's dream to family, friends, or a partner), and dream recording frequencies are self-reported and reflect typical habits over the past year. Percentages are based on valid responses (EFA: *n* = 432; CFA: *n* = 211).

The recruitment email outlined the aim of the study, the eligibility criteria, and the instruction to complete the survey immediately upon waking. The online form opened with a written page describing the study and restating these conditions, followed by the informed consent form. Participants then completed the sociodemographic questionnaire and the sleep-related questions (see Table 1). A written instruction page for the CSCS-REM was presented next, explaining the comparative response format and providing an illustrative example (see Appendix A and B). Participants were then asked whether they recalled their most recent dream and how much time had elapsed since it occurred. Those who did not recall a dream were thanked and informed that they could return on another day when they had a recalled dream. Completion of the study took 20 minutes. The study was approved by the Non-Interventional Clinical Research Ethics Committee of Istanbul Medipol University (Approval No: 10840098-772.02-5296).

### Instruments

The Bodily Self-Consciousness in Dreams Questionnaire (BSD-Q) and the Dream Reflective Awareness Questionnaire (DRAQ) were administered to assess the convergent validity of the scale. The Consciousness States Comparison Scale: REM Version (CSCS-REM) was developed through EFA with subsequent reliability and validity testing, and its factor structure was further examined by CFA on an independent sample.

#### Participant information form

A form developed by the researchers was used to collect data on participants’ sex, educational attainment, history of neurological or psychiatric disorders, regular medication use, diagnoses of sleep disorders, and use of sleep medication. Additional information was obtained regarding average sleep duration and the frequency of sharing or recording dreams. Prior to completing the CSCS-REM, participants were asked whether they recalled their most recent dream and how much time had elapsed since it occurred. These data were used to assess whether participants met the inclusion and exclusion criteria.

#### Bodily self-consciousness in dreams questionnaire

The BSD-Q is a 12-item, 7-point Likert-type self-report scale developed by Erdeniz et al. (2023) to assess bodily awareness during dreams across four subdimensions: proprioceptive, somatosensory, vestibular, and visual modalities. Its four-factor structure accounted for 64% of the total variance, with internal consistency coefficients ranging from Cronbach’s α = .61–.84 and McDonald’s ω = .62–.84. In the present study, the BSD-Q was administered to measure participants’ levels of bodily sensory awareness in relation to their most recently recalled dream experiences.

#### Dream reflective awareness questionnaire

The DRAQ, developed by Lee et al. (2007), is a 19-item, 5-point Likert-type scale (0–4) that measures reflective self-awareness in dreams across five subdimensions: Lucid Mindfulness, Dual Perspectives, Depersonalization, Intra-dream Self-awareness, and Willed Appearances. Its Turkish adaptation was carried out by Genç et al. (2016). A validation study confirmed the five-factor structure, which explained 71% of the total variance, with Cronbach’s α coefficients ranging from .74 to .78. In the present study, the DRAQ was used to evaluate participants’ reflective self-awareness in their most recently recalled dreams.

#### *Consciousness states comparison scale: REM version (CSCS-REM,* provided in the appendices)

The CSCS-REM was developed to assess participants’ phenomenological experiences in REM dreams by comparing them with waking consciousness. The scale consists of 35 items, each rated on a 6-point Likert-type scale ranging from 0 to 5 (0 = no response/no experience, 1 = very low, 2 = low, 3 = same as waking, 4 = high, 5 = very high). Exploratory factor analysis identified eight subdimensions: Metacognitive Awareness (10 items: 8–17), Cognitive Fluency (5 items: 18–22), Spatial Awareness (3 items: 30–32), Temporal Awareness (4 items: 26–29), Emotional Awareness (3 items: 33–35), Memory (3 items: 23–25), Interoceptive and Chemical Senses (3 items: 1–2, 7), and Perceptual and Bodily Senses (4 items: 3–6). The scale yields both subdimension scores and a total score, which can be computed as either sum or mean values. For comparisons across subdimensions, the use of mean scores is recommended. A methodological caveat, however, is that mean values may conflate zeros, which indicate the absence of experience, with low ratings (1–2), which indicate weak experience. This conflation limits the ability to distinguish between non-occurrence and low intensity. Accordingly, depending on the research objective, either sum or mean scoring may be applied. When interpreting mean scores, values approximating 3 reflect equivalence with waking experience, values above 3 reflect higher levels relative to waking, and values below 3 reflect lower levels. A score of 0 indicates that the experience was absent or not reported. Zeros can also be analyzed separately, for instance through frequency distributions, to capture the absence of specific contents. If zeros are excluded, a response validity threshold (*e.g.* at least 70% of items completed) should be established.

### Statistical analyses

EFA was conducted on data from 432 participants, and CFA on data from 211 participants. Prior to analyses, data integrity and item adequacy were examined. All corrected item-total correlations exceeded .30, and Cronbach’s α remained stable across item deletions. Descriptive statistics and percentage distributions of the participants’ demographic and clinical characteristics were calculated. The “No response” option (scored as 0) was treated as a valid response indicating the absence of phenomenological experience, not as missing data, and was included in all analyses accordingly.

For the EFA, sample adequacy was evaluated through the Kaiser-Meyer-Olkin test and Bartlett’s test of sphericity. Factor extraction was carried out using the Maximum Likelihood method, and Oblimin oblique rotation was applied to allow factor intercorrelations. The number of factors was determined using multiple criteria: the eigenvalue > 1 rule, parallel analysis, inspection of the scree plot, and the proportion of explained variance. Items with low factor loadings or high cross-loadings were excluded. The remaining items were labeled according to the factor structure, and internal consistency for each subscale was estimated using both Cronbach’s α and McDonald’s ω coefficients. Discriminant validity was evaluated using the Fornell-Larcker criterion. The square root of the AVE for each factor was required to exceed the inter-factor correlations. Independent samples t-tests were conducted on subscale mean scores for the upper and lower 27% groups. For criterion validity, correlations between the developed scale and the subdimensions of the BSD-Q and DRAQ scales were examined using Pearson’s correlation coefficients. CFA was conducted to test the eight-factor model derived from the EFA on an independent sample. The Diagonally Weighted Least Squares estimation method was employed. Model fit was evaluated using χ^2^/df, Comparative Fit Index, Tucker-Lewis Index, Normed Fit Index (NFI), Incremental Fit Index (IFI), Relative Fit Index (RFI), Parsimony Normed Fit Index (PNFI), Standardized Root Mean Square Residual (SRMR), and Root Mean Square Error of Approximation (RMSEA). The significance of all standardized factor loadings was assessed. All analyses were performed using SPSS, Jamovi, and R software.

## Results

### Exploratory factor analysis and reliability-validity analysis

Univariate outliers were examined through standardized z-scores (|Z| > 3.29), and multivariate outliers were assessed using Mahalanobis distances (χ^2^, *P* < .001 threshold). Item variances were deemed adequate, and corrected item-total correlations ranged between .317 and .665, all exceeding the .30 threshold. The Cronbach’s α coefficient was .962, and the removal of any item did not meaningfully increase this value. Prior to EFA, frequency analyses were conducted to examine the distribution of zero responses across subscales. Zero-response rates were lowest for Emotional Awareness (3.6%–7.8%) and Cognitive Fluency (3.8%–10.5%), followed by Perceptual and Bodily Senses (4.7%–15.0%), Spatial Awareness (5.4%–16.6%), Metacognitive Awareness (6.3%–19.7%), and Memory (5.8%–28.0%). Higher zero-response rates were observed for Temporal Awareness (14.3%–28.0%) and Interoceptive and Chemical Senses (29.8%–54.5%).

The Kaiser-Meyer-Olkin measure of sampling adequacy (.921) and Bartlett’s test of sphericity, χ^2^(595) = 7852.51, *P* < .001, confirmed that the data were suitable for factor analysis. Factors were extracted using the Maximum Likelihood method with Oblimin oblique rotation. According to the eigenvalue > 1 criterion, eight factors were identified, explaining 56.04% of the total variance. This solution was further supported by Horn's parallel analysis (FA-based), which confirmed that eight factors from the observed data exceeded the corresponding eigenvalues generated from simulated random data. Visual inspection of the scree plot was also consistent with an eight-factor solution. The model’s goodness-of-fit test was significant, χ^2^(343) = 751.20, *P* < .001. Items with low loadings (< .30) or substantial cross-loadings (> .35) were eliminated, resulting in a final scale of 35 items organized under eight factors. Inter-factor correlations ranged from .122 to .581, suggesting that while the factors were moderately correlated, they retained sufficient distinctiveness to be considered separable constructs. Based on their loadings, the factors were designated as Metacognitive Awareness, Emotional Awareness, Spatial Awareness, Interoceptive and Chemical Senses, Perceptual and Bodily Senses, Memory, Cognitive Fluency, and Temporal Awareness.

In the final version of the scale, consisting of 35 items retained after the exploratory factor analysis, all items loaded above the minimum acceptable threshold of .30, indicating a generally clean and well-defined factor structure (Table 2). A few items (*e.g.* CSCS-REM_18, CSCS-REM_3, CSCS-REM_4, and CSCS-REM_23) showed comparatively weaker loadings, with CSCS-REM_4 also displaying a secondary loading on the Interoceptive and Chemical Senses factor. Although this pattern represents cross-loading methodologically, it was deemed theoretically acceptable given the item’s direct assessment of tactile perception, which is conceptually relevant to both factors. For this reason, the item was retained to preserve the theoretical integrity of the construct and was carried forward to confirmatory factor analysis for further evaluation.

**Table 2:** Exploratory factor analysis of the eight-factor model: Factor loadings

Factor	1	2	3	4	5	6	7	8
Factor 1: metacognitive awareness								
CSCS-REM_9	**.77398**	.01222	.06738	.03544	−.04124	−.04493	−.04895	.04859
CSCS-REM_10	**.69645**	−.09333	−.02049	.00473	.22322	−.00558	−.05032	.04335
CSCS-REM_11	**.68821**	−.05296	.04596	.0381	.01804	.10488	−.05349	.07983
CSCS-REM_8	**.67092**	.02878	.06654	.04475	−.08662	−.01534	.12309	.0177
CSCS-REM_17	**.62855**	.19349	.02737	.07938	−.06727	−.06638	.04353	.03037
CSCS-REM_15	**.61195**	.08887	.02806	.06369	.10748	−.03166	.02576	−.02783
CSCS-REM_12	**.59636**	.09899	−.03604	−.03158	.06226	.14275	−.00278	.00205
CSCS-REM_14	**.54624**	.09836	.08549	.04063	.09468	−.06157	.04851	−.03377
CSCS-REM_13	**.4991**	.10486	−.00665	−.02323	−.05036	.21913	.18985	−.0624
CSCS-REM_16	**.48697**	.16391	−.04692	−.00553	.08053	.09522	.00521	.03566
Factor 2: cognitive fluency								
CSCS-REM_21	−.04457	**.80397**	.06191	.00131	.02396	.04866	.01381	−.00199
CSCS-REM_22	.09227	**.7277**	.02815	.11477	−.01466	.0435	.00785	−.07863
CSCS-REM_19	.04424	**.57917**	.03781	−.00806	.04327	−.02017	−.06374	.20897
CSCS-REM_20	.22631	**.51953**	−.02721	−.02675	.14414	.06535	.02568	−.04032
CSCS-REM_18	.08874	**.33399**	−.00917	.13209	.02025	−.03243	.04769	.16366
Factor 3: spatial awareness								
CSCS-REM_31	−.01842	.01561	**.91631**	−.01759	.01418	−.02042	.0142	−.01073
CSCS-REM_32	.06585	−.00562	**.69793**	−.00662	.09205	.0692	.06579	−.06251
CSCS-REM_30	.03242	.05781	**.64227**	.09897	−.0398	.00538	−.07166	.16174
Factor 4: temporal awareness								
CSCS-REM_26	−.03811	.04135	.01003	**.82008**	.04559	−.03963	−.02249	.00644
CSCS-REM_27	.10201	.03192	−.02521	**.70773**	.04633	.04539	.03274	.0269
CSCS-REM_28	.0183	−.00837	.14566	**.42818**	−.06172	.29904	.03159	.01126
CSCS-REM_29	.09448	−.09937	.20891	**.40506**	−.05008	.19375	.13172	−.07948
Factor 5: emotional awareness								
CSCS-REM_33	.00565	.01083	.02785	.0484	**.92416**	−.02288	.02475	−.00549
CSCS-REM_34	−.00408	.01143	−.00339	.0117	**.56121**	.08033	−.05578	.02791
CSCS-REM_35	.06722	.0708	.0979	−.03905	**.54649**	.07126	.0068	.09087
Factor 6: Memory								
CSCS-REM_24	−.0197	.02661	.00333	−.00746	.01557	**.85874**	.01774	.02908
CSCS-REM_25	.01798	.06544	.04201	.10433	−.02074	**.6405**	.0468	.0096
CSCS-REM_23	.08758	.0841	.13353	.04539	.15248	**.38603**	−.07105	.11814
Factor 7: interoceptive and chemical senses								
CSCS-REM_2	−.03916	.01104	.03895	−.00419	.00985	−.02031	**.79199**	.04089
CSCS-REM_1	.00554	−.01198	.00499	.04988	−.0193	.05394	**.71686**	.000487
CSCS-REM_7	.19562	−.09137	.01852	−.0245	.07376	.1421	**.46837**	−.0731
Factor 8: perceptual and bodily senses								
CSCS-REM_5	.03365	−.03345	.00238	.04897	−.00696	.05732	.02085	**.82165**
CSCS-REM_6	.05859	.0286	.0957	−.06891	.0695	.01037	−.03118	**.59924**
CSCS-REM_4	.00145	.08989	−.00452	−.04299	.15378	−.04935	.36448	**.39586**
CSCS-REM_3	−.00808	.12075	−.04885	.02081	.03831	−.08782	.27309	**.3725**

*Note.* Maximum likelihood extraction method with oblimin rotation was applied. Bold values indicate the primary factor loading for each item.

The final scale structure exhibited satisfactory to high levels of internal consistency across all subdimensions. Cronbach’s α coefficients ranged from .736 to .915, while McDonald’s ω coefficients ranged from .639 to .901 (Table 3).

**Table 3:** Internal consistency reliability coefficients for the total scale and its subscales

Scale/Subscale	Cronbach's α	ω (McDonald’s)	Number of items
Total scale	.939	.951	35
Metacognitive awareness	.915	.901	10
Spatial awareness	.846	.848	3
Cognitive fluency	.836	.820	5
Temporal awareness	.820	.786	4
Emotional awareness	.778	.703	3
Memory	.773	.774	3
Interoceptive and Chemical Senses	.773	.639	3
Perceptual and Bodily Senses	.736	.799	4

*Note.* Values represent internal consistency coefficients for the total scale and each subscale of the Consciousness States Comparison Scale: REM Version (CSCS-REM). All coefficients indicate acceptable to excellent reliability across dimensions.

To evaluate discriminant capacity, independent samples t-tests were conducted for each subdimension. As shown in Table 4, statistically significant differences (*P* < .001) were observed between the lower 27% and upper 27% groups across all subdimensions. In every case, mean scores of participants in the upper 27% group were higher than those in the lower 27% group. The largest mean difference emerged in the Interoceptive and Chemical Senses subdimension (Upper group M = 2.82, Lower group M = .06), followed by Memory (M = 3.72 versus .79) and Spatial Awareness (M = 4.05 versus .96). Similar patterns were found in all other subdimensions, consistently reflecting large effect sizes.

**Table 4:** Independent samples t-tests for scale subdimensions (lower versus upper 27% groups)

Subdimension	Lower 27% (*n* = 117) M (SD)	Upper 27% (*n* = 117) M (SD)	*t*	df	95% CI [LL, UL]
Perceptual and bodily senses	1.62 (0.76)	3.87 (0.44)	−27.78^***^	186.77	[−2.41, −2.09]
Interoceptive and chemical senses	0.06 (0.13)	2.82 (0.56)	−52.15^***^	127.73	[−2.87, −2.66]
Temporal awareness	0.63 (0.36)	3.20 (0.55)	−41.55^***^	202.37	[−2.70, −2.45]
Cognitive fluency	1.48 (0.63)	3.82 (0.49)	−31.60^***^	218.95	[−2.48, −2.19]
Memory	0.79 (0.49)	3.72 (0.56)	−42.59^***^	232	[−3.07, −2.80]
Spatial awareness	0.96 (0.56)	4.05 (0.59)	−40.95^***^	232	[−3.24, −2.94]
Emotional awareness	1.74 (0.82)	4.42 (0.42)	−31.59^***^	172.28	[−2.85, −2.52]
Metacognitive awareness	1.29 (0.57)	3.75 (0.46)	−36.50^***^	222.52	[−2.59, −2.33]

*Note.* Values represent group means and standard deviations for lower 27% (*n* = 117) and upper 27% (*n* = 117) scorers. Negative t values reflect the comparison direction (Lower group as reference); the magnitude of t indicates the strength of the group difference. CI = confidence interval; LL = lower limit; UL = upper limit. *P* < .05 = ^*^  *P* < .01 = ^**^  *P* < .001 = ^***^

The Fornell-Larcker discriminant validity assessment demonstrated that the square root of the AVE for each factor exceeded its correlations with all other constructs, supporting the discriminant validity of the eight-factor structure (Table 5). The strongest inter-factor correlation was observed between Metacognitive Awareness and Cognitive Fluency (*r* = .582).

**Table 5:** Fornell-Larcker discriminant validity analysis for the eight-factor solution

Factor	1	2	3	4	5	6	7	8
1. Metacognitive awareness	**.626**	.466	.412	.235	.374	.582	.284	.439
2. Emotional awareness	.466	**.699**	.368	.139	.156	.345	.351	.202
3. Spatial awareness	.412	.368	**.761**	.269	.415	.393	.261	.507
4. Interoceptive and Chemical Senses	.235	.139	.269	**.674**	.343	.182	.205	.204
5. Memory	.374	.156	.415	.343	**.658**	.327	.121	.434
6. Cognitive fluency	.582	.345	.393	.182	.327	**.615**	.233	.398
7. Perceptual and Bodily Senses	.284	.351	.261	.205	.121	.233	**.577**	.131
8. Temporal awareness	.439	.202	.507	.204	.434	.398	.131	**.617**

*Note.* Bolded diagonal values represent the square root of the Average Variance Extracted (AVE) for each construct. Off-diagonal values represent the latent construct correlations. Discriminant validity is supported when each diagonal value exceeds the corresponding off-diagonal correlations in its row and column (Fornell and Larcker 1981).

All eight subdimensions showed significant positive associations with BSD-Q and DRAQ subdimensions. The strongest effects emerged for Memory (Lucid Mindfulness, *r* = .348, *P* < .01; Intra-dream Self-awareness, r = .327, *P* < .01) and Metacognitive Awareness (Intra-dream Self-awareness, *r* = .353, *P* < .01). Other subdimensions yielded small-to-moderate correlations with relevant BSD-Q sensory subdimensions (*e.g.* Somatosensory, Proprioceptive) and DRAQ reflective subdimensions (*e.g.* Dual Perspectives, Willed Appearances). All correlation coefficients and significance levels are reported in Table 6.

**Table 6:** Pearson correlations between CSCS-REM subdimensions and convergent validation measures (BSD-Q and DRAQ)

	1	2	3	4	5	6	7	8	9	10	11	12	13	14	15	16	17
1. Perceptual and bodily senses	—																
2. Interoceptive and chemical senses	.544^**^	—															
3. Temporal awareness	.226^**^	.544^**^	—														
4. Cognitive fluency	.365^**^	.247^**^	.544^**^	—													
5. Memory	.281^**^	.371^**^	.569^**^	.460^**^	—												
6. Spatial awareness	.329^**^	.302^**^	.562^**^	.465^**^	.498^**^	—											
7. Emotional awareness	.400^**^	.202^**^	.280^**^	.441^**^	.325^**^	.419^**^	—										
8. Metacognitive awareness	.392^**^	.333^**^	.521^**^	.677^**^	.508^**^	.493^**^	.506^**^	—									
9. BSD-Q vestibular modality	.086	.169^**^	.113^*^	.111^*^	.140^**^	.016	.106^*^	.097	—								
10. BSD-Q somatosensory modality	.293^**^	.207^**^	.129^*^	.202^**^	.132^*^	.113^*^	.258^**^	.191^**^	.349^**^	—							
11. BSD-Q visual modality	.033	.156^**^	.116^*^	.033	.095	.033	.092	.056	.452^**^	.273^**^	—						
12. BSD-Q proprioceptive modality	.273^**^	.262^**^	.208^**^	.175^**^	.151^**^	.147^**^	.193^**^	.196^**^	.455^**^	.447^**^	.490^**^	—					
13. DRAQ lucid mindfulness	.053	.252^**^	.255^**^	.134^*^	.348^**^	.189^**^	.040	.190^**^	.126^*^	.023	.107^*^	.084	—				
14. DRAQ dual perspectives	.144^**^	.187^**^	.227^**^	.130^*^	.255^**^	.177^**^	.122^*^	.196^**^	.226^**^	.146^**^	.189^**^	.243^**^	.360^**^	—			
15. DRAQ depersonalization	.070	.275^**^	.211^**^	.049	.278^**^	.124^*^	.087	.094	.225^**^	.134^*^	.289^**^	.283^**^	.361^**^	.501^**^	—		
16. DRAQ intra-dream self-awareness	.109^*^	.223^**^	.302^**^	.257^**^	.327^**^	.263^**^	.236^**^	.353^**^	.127^*^	.227^**^	.177^**^	.189^**^	.429^**^	.320^**^	.283^**^	—	
17. DRAQ willed appearances	.061	.289^**^	.281^**^	.250^**^	.300^**^	.230^**^	.095	.278^**^	.195^**^	.165^**^	.282^**^	.180^**^	.484^**^	.394^**^	.447^**^	.502^**^	—

*Note.* Values represent Pearson correlation coefficients. Asterisks indicate significance levels after controlling for multiple comparisons using the Benjamini-Hochberg False Discovery Rate (FDR) correction: *P* < .05 = ^*^  *P* < .01 = ^**^  *P* < .001 = ^***^. All significant correlations reported in the table remained significant after applying the FDR adjustment, indicating that the observed associations are robust and not due to inflated Type I error from multiple testing. BSD-Q = *Bodily Self-Consciousness in Dreams Questionnaire*; DRAQ = *Dream Reflective Awareness Questionnaire.*

### Confirmatory factor analysis

CFA was conducted on an independent sample. Of the 309 participants initially reached, those who reported not recalling a dream, had neurological or psychiatric diagnoses, were using medication, or failed the attention-check item were excluded, yielding a final sample of 211 participants. Demographic, sleep, and dream-related characteristics of this sample are presented in Table 1.

The CFA results showed that the proposed model demonstrated a good to excellent fit to the observed data, χ^2^(532) = 925.00, *P* < .001, χ^2^/df = 1.74, CFI = .986, TLI = .984, NFI = .967, IFI = .986, RFI = .963, PNFI = .865, SRMR = .069, RMSEA = .059 (90% CI [.053, .066], *P* = .009). All standardized factor loadings ranged from .489 to .877 and were significant at the *P* < .001 level.

The CFA results showed that all items in the measurement model loaded significantly and at adequate levels on their respective factors (*P* < .001). Standardized loadings for the Metacognitive Awareness factor ranged from .657 to .859. For Emotional Awareness, loadings ranged from .489 to .831; for Spatial Awareness (SA), from .787 to .877; for Interoceptive and Chemical Senses, from .703 to .742; for Memory, from .746 to .801; for Cognitive Fluency, from .554 to .802; for Perceptual and Bodily Senses, from .643 to .847; and for Temporal Awareness, from .720 to .807. Overall, all items showed factor loadings above the acceptable threshold of .40, thereby supporting the validity of the measurement model (Table 7).

**Table 7:** Standardized factor loadings, standard errors, z-values, and significance levels for the measurement model

Factor	Item	β	SE	z	*P*
Metacognitive awareness	CSCS-REM_8	.692	—	—	—
Metacognitive awareness	CSCS-REM_9	.859	.0380	32.7	<.001
Metacognitive awareness	CSCS-REM_10	.800	.0357	32.4	<.001
Metacognitive awareness	CSCS-REM_11	.705	.0339	30.0	<.001
Metacognitive awareness	CSCS-REM_12	.759	.0353	31.1	<.001
Metacognitive awareness	CSCS-REM_13	.657	.0346	27.4	<.001
Metacognitive awareness	CSCS-REM_14	.669	.0333	29.1	<.001
Metacognitive awareness	CSCS-REM_15	.704	.0338	30.1	<.001
Metacognitive awareness	CSCS-REM_16	.713	.0353	29.2	<.001
Metacognitive awareness	CSCS-REM_17	.766	.0357	31.0	<.001
Emotional awareness	CSCS-REM_33	.789	—	—	—
Emotional awareness	CSCS-REM_34	.489	.0388	16.0	<.001
Emotional awareness	CSCS-REM_35	.831	.0517	20.3	<.001
Spatial awareness	CSCS-REM_30	.787	—	—	—
Spatial awareness	CSCS-REM_31	.877	.0464	24.0	<.001
Spatial awareness	CSCS-REM_32	.860	.0428	25.5	<.001
Interoceptive and chemical senses	CSCS-REM_1	.703	—	—	—
Interoceptive and chemical senses	CSCS-REM_2	.729	.0667	15.5	<.001
Interoceptive and chemical senses	CSCS-REM_7	.742	.0623	16.9	<.001
Memory	CSCS-REM_23	.747	—	—	—
Memory	CSCS-REM_24	.746	.0423	23.6	<.001
Memory	CSCS-REM_25	.801	.0451	23.8	<.001
Cognitive fluency	CSCS-REM_18	.554	—	—	—
Cognitive fluency	CSCS-REM_19	.717	.0577	22.4	<.001
Cognitive fluency	CSCS-REM_20	.802	.0612	23.7	<.001
Cognitive fluency	CSCS-REM_21	.788	.0612	23.2	<.001
Cognitive fluency	CSCS-REM_22	.773	.0603	23.2	<.001
Perceptual and bodily senses	CSCS-REM_3	.643	—	—	—
Perceptual and bodily senses	CSCS-REM_4	.804	.0780	16.0	<.001
Perceptual and bodily senses	CSCS-REM_5	.847	.0784	16.8	<.001
Perceptual and bodily senses	CSCS-REM_6	.710	.0679	16.3	<.001
Temporal awareness	CSCS-REM_26	.720	—	—	—
Temporal awareness	CSCS-REM_27	.807	.0461	24.3	<.001
Temporal awareness	CSCS-REM_28	.774	.0433	24.8	<.001
Temporal awareness	CSCS-REM_29	.747	.0433	23.9	<.001

*Note. Item* = observed variable code; *β* = standardized factor loading; *SE* = standard error; *z* = z-statistic; *P* = significance level. All loadings are significant at *P* < .001.

Table 8 presents the factor correlation matrix for the measurement model. Positive and significant associations were observed among all constructs (*P* < .01). All correlation values remained below the commonly accepted threshold of *r* = .85 used in discriminant validity assessments. Thus, although some factor pairs show conceptual closeness, no critical risk of multicollinearity is present. Overall, the correlation structure supports the hypothesized measurement model and demonstrates that inter-factor relationships are theoretically interpretable.

**Table 8:** Factor correlation matrix for the measurement model

	1	2	3	4	5	6	7	8
1. Metacognitive awareness	—							
2. Emotional awareness	.544^**^	—						
3. Spatial awareness	.466^**^	.338^**^	—					
4. Interoceptive and chemical senses	.507^**^	.344^**^	.298^**^	—				
5. Memory	.568^**^	.557^**^	.525^**^	.592^**^	—			
6. Cognitive fluency	.804^**^	.566^**^	.413^**^	.497^**^	.577^**^	—		
7. Perceptual and bodily senses	.345^**^	.382^**^	.315^**^	.421^**^	.289^**^	.421^**^	—	
8. Temporal awareness	.606^**^	.413^**^	.598^**^	.648^**^	.712^**^	.574^**^	.246^**^	—

*Note.* Values represent standardized correlations between latent constructs. Diagonal values are fixed at 1.00. P < .05 = ^*^ P < .01 = ^**^ P < .001 = ^***^

## Discussion

This study introduces a novel self-report scale designed to compare REM dream consciousness with waking consciousness. The findings provide evidence for the scale’s construct validity and reliability, thereby addressing a critical methodological gap in consciousness research. Whereas existing instruments typically focus either exclusively on dream experiences or on isolated dimensions of dreaming, the present scale enables a direct comparison between dream and waking states within a single, standardized format.

The EFA yielded an eight-factor solution comprising Metacognitive Awareness, Emotional Awareness, Spatial Awareness, Interoceptive and Chemical Senses, Perceptual and Bodily Senses, Memory, Cognitive Fluency, and Temporal Awareness. The eight-factor solution captures the richness of REM consciousness across content and experience. The factors are distinct, with each representing a separable phenomenological domain, yet moderately correlated (see Table 8). This pattern is consistent with contemporary neurocognitive perspectives, which emphasize that dream consciousness can differentiate from waking consciousness across specific dimensions, with each dimension contributing uniquely to phenomenological experience (*e.g.*  Hobson 2009; Tononi et al. 2024). By enabling participants to report phenomenological similarities and differences between REM dreaming and waking consciousness across emotional, sensory, and cognitive domains, the scale makes continuity and discontinuity claims (*e.g.*  Nir and Tononi 2010; Horton 2017) empirically testable and, unlike previous approaches, allows their evaluation within a single psychometric construct. The eight-factor solution, which accounted for 56.04% of the variance, captures the phenomenological diversity of dream consciousness. Cronbach's alpha and McDonald's ω values confirm the internal consistency of all subdimensions.

Metacognitive Awareness emerged as one of the most prominent subscales of the CSCS-REM. Prior studies propose that meta-awareness and metacognitive functions are suppressed during REM sleep (Cicogna and Bosinelli 2001; Gott et al. 2024). This subdimension offers a strong basis for examining that theoretical claim through subjective report. The Cognitive Fluency subscale, in turn, comprises items that assess the online functioning of mental processes such as sustaining attention and producing fluent speech. The findings indicate that although dreams often contain logical discontinuities and fragmented narratives (Sutton et al. 1994), these cognitive processes are frequently experienced as fluent at the subjective level (Kahan et al. 1997). Previous studies report that unrecognized cognitive inconsistencies in dreams reflect metacognitive suppression (Rechtschaffen 1978; Kahan and LaBerge 2011), while basic cognitive processes such as speech, attention, or problem-solving attempts may continue with a degree of fluency (Meier 1993; Kahan et al. 1997). Thus, the Cognitive Fluency subscale appears to tap the execution of cognitive functions within dreams, while the Metacognitive Awareness subscale appears to tap awareness and regulation of those functions.

The separation of sensory modalities into internal/chemical (taste, smell, interoceptive sensations) and external/bodily (touch, body image) subscales provides evidence for the multimodal nature of dream consciousness. This finding accords with the view that dreams constitute weakly phenomenally functional embodied states, connected to a phenomenal self-derived from waking experience (Thompson 2015; Windt 2020). The notion advanced by Revonsuo (1995, 2006) and Hobson (2009) that dreams constitute a dynamic and multimodal world simulation also supports this distinction. The emergence of external/bodily senses as a separate factor indicates continuity between dreams and the bodily self-experienced in wakefulness, whereas the separation of internal/chemical senses as an independent factor suggests that internal homeostatic processes also contribute to the construction of dream consciousness. Wamsley (2013) noted that the phenomenology of dreams is grounded in the sensory modalities active during wakefulness, yet these modalities are reorganized in a different form within dreaming. In our sample, the reorganization took a particular shape. In waking perception, sensory modalities typically differentiate into separate subdimensions such as vision, audition, touch, taste, and smell. In our data, these modalities did not form separate subscales; they clustered into two broader groups along an internal-external axis (interoceptive/chemical versus perceptual/bodily). Several interpretations of this pattern are possible. The clustering may reflect how dream experience itself is organized, with the internal-external distinction carrying more variance than modality-specific distinctions. It may also reflect processes at the level of recall, in which modality-specific detail becomes less distinguishable during retrospective report. A further possibility is that the comparative scoring format of the CSCS-REM attenuates modality-specific variance, since items are rated relative to waking experience. These alternatives cannot be disentangled from the present data and warrant examination in future work. Within this factor structure, the tactile experience item displayed cross-loading between the Perceptual/Bodily and Interoceptive/Chemical factors. The decision to retain the item reflected a balance between theoretical coherence and statistical adequacy: excluding it would reproduce a gap already recognized in the field. Tactile and bodily experiences are underrepresented in dream reports themselves (Okada et al. 2005) and the same gap is present in the instruments used to assess dream and consciousness phenomenology, which motivated the development of dedicated scales such as the BSD-Q (Erdeniz et al. 2023).

The Spatial Awareness subscale was defined by items with high loadings (*e.g.* .92, .70, .64), capturing the relation of dreams to bodily positioning and spatial context. This corresponds with Revonsuo’s (2006) account of dreams as a ‘world simulation.’ Themes such as flying, falling, or spatial navigation are frequently reported (Nielsen et al. 2003; Maggiolini et al. 2007; Schredl and Piel 2007), and together with visual-vestibular content, indicate that spatial awareness constitutes a core dimension of dream consciousness. By contrast, the Temporal Awareness subscale was characterized by comparatively lower and more dispersed loadings (.82 to .41), suggesting that temporal perception represents a more fragile and variable dimension in dreams. This pattern accords with phenomenological observations that dream experience often lacks temporal continuity, with events unfolding in sequences that violate chronological order (Sutton et al. 1994). Reports of time dilation and contraction in dreams further align with this interpretation (Erlacher et al. 2014).

The Memory factor comprised three items capturing different types of memory contents: personally experienced events (Item 23), historical events and facts (Item 24), and newly learned information (Item 25). Memory has long been recognized as a component of dream experience, with research linking dreaming to consolidation processes and the reactivation of waking memory traces (Fosse et al. 2003; Wamsley et al. 2010a, 2010b; Domhoff 2011). Two lines of evidence map onto the continuity and discontinuity hypotheses. On the continuity side, the presence of memory content in dreams is in line with the classical day-residue effect, according to which recent waking experiences are incorporated into dreams within a day or two (Freud 1953), and with the dream-lag effect, according to which waking life events reappear in dreams after a delay of 5 to 7 days (Nielsen et al. 2004). On the discontinuity side, dream cognition research has reported that recalling, manipulating, and accessing memory contents during dreams is often weaker than in wakefulness, with limited retrieval of autobiographical episodes and reduced access to factual knowledge (Hartmann 2000; Hobson et al. 2000). The Memory factor of the CSCS-REM, by indexing how memory-related experiences in dreams compare with their waking counterparts, may inform future studies examining how these patterns unfold across individuals and dream conditions.

The Emotional Awareness subscale, defined by high item loadings, indicates that dreams are interwoven not only with cognitive contents but also with strong affective experiences. Previous studies have shown that dream reports frequently involve intense emotions and that these emotions shape the structure of dream experience (Fosse et al. 2001; Schredl and Reinhard 2010). Furthermore, the emotional tone of dreams carries over into subsequent waking mood; negative dreams can disrupt daytime emotional stability, whereas positive dreams have been reported to exert a regulatory effect (Cartwright et al. 2006).

The correlations among factors reflect the theoretically expected proximities. The strong correlation between Metacognitive Awareness and Cognitive Fluency indicates that, although the two subscales are functionally distinguishable, they remain theoretically related. Similarly, the robust association between Memory and Temporal Awareness reflects the reorganization of past experiences into dream narratives within a temporal framework. The moderate correlation between Emotional Awareness and Memory is consistent with the view that dreams contribute to emotional regulation and that emotions mediate the processing of information in memory (Cartwright 2011). However, emotionally intense experiences are known to be associated with higher memorability (Kensinger 2009). Accordingly, the greater ease of recalling emotionally charged dreams may imply that the observed correlation partly arises from recall bias.

Comparisons between the upper and lower 27% groups revealed significant differences across all subscales, supporting discriminant validity and sensitivity to individual differences in conscious experience. The Fornell-Larcker analysis demonstrated full discriminant validity, with the square root of AVE for each factor exceeding all of its inter-factor correlations. Although AVE values ranged from .333 for Perceptual and Bodily Senses to .579 for Spatial Awareness, with several falling below the conventional .50 threshold, all subscales satisfied the alternative criterion proposed by Fornell and Larcker (1981) for cases of low AVE: composite reliability above .60. McDonald's ω ranged from .639 to .901 across the eight subscales, supporting discriminant validity through this fallback rule. The strongest inter-factor correlation emerged between Metacognitive Awareness and Cognitive Fluency (*r* = .582). Metacognitive Awareness encompasses the awareness, monitoring, and control of cognitive processes, whereas Cognitive Fluency reflects the online execution of those processes, such as sustained attention and fluent language use. Previous studies have emphasized that the efficiency of cognitive functioning depends largely on metacognition (Koriat 2007; Efklides 2008). A complementary relationship therefore exists between the two: the quality of cognitive performance shapes the effectiveness of metacognitive processes, while metacognitive regulation allows cognitive processes to be deployed in a flexible, purposeful, and efficient manner. The empirical proximity observed between these subscales is consistent with their theoretical interrelation within conscious experience. It should also be noted that the Fornell-Larcker criterion has been shown to perform less well in detecting subtle discriminant validity issues when inter-factor correlations are high (Henseler et al. 2015); although the criterion was satisfied in the present analysis, future research could complement it with the heterotrait-monotrait ratio for more sensitive evidence, and could also examine whether Metacognitive Awareness and Cognitive Fluency might be represented under a higher-order factor through bifactor or second-order CFA modeling. Such modeling would preserve the theoretical distinction between awareness of cognition and execution of cognition while accounting for their close empirical relationship.

Correlation analyses with the BSD-Q and DRAQ subscales provided evidence for convergent validity. The Perceptual and Bodily Senses subscale was significantly related to the Somatosensory and Proprioceptive subscales of the BSD-Q, indicating that bodily awareness is adequately represented. It also showed small but significant associations with the DRAQ subscales of Dual Perspectives and Intra-dream Self-Awareness, suggesting a contribution of bodily perception to reflective self-awareness. The Interoceptive and Chemical Senses subscale correlated significantly with all subscales of both instruments. The positive associations with the BSD-Q may reflect the unity of phenomenological experience, cross-modal integration, and the shared neural substrates of bodily representation. The correlations with the DRAQ, in turn, may suggest that interoceptive and visceral awareness contributes to higher-order reflective processes in dreaming. Similarly, the Temporal Awareness subscale was positively correlated with all subscales of the BSD-Q and the DRAQ. Spatial Awareness showed only limited overlap with the BSD-Q, which may suggest that spatial experience in dreams is partly related to bodily awareness. In addition, the significant associations with the DRAQ subscales of Intra-dream Self-Awareness and Willed Appearances may imply that spatial referencing plays a role in establishing the subject-environment distinction during dreaming. As expected, the Emotional Awareness subscale showed significant correlations with the vestibular, somatosensory, and proprioceptive modalities of the BSD-Q, which may lend support to the view that emotional processes experienced in dreams are connected to bodily sensory awareness. Memory, Cognitive Fluency, and Metacognitive Awareness exhibited their strongest correlations with the DRAQ subscales of Intra-dream Self-Awareness and Willed Appearances. This pattern may point to the possibility that cognitive processes contribute to meta-representational capacity and voluntary actions in dreams.

Overall, comparisons with the BSD-Q and DRAQ subscales yielded significant but generally small-to-moderate correlations across nearly all dimensions. The CSCS-REM’s reweighting approach, based on differences relative to waking consciousness, may not capture exactly the same latent constructs as instruments assessing absolute intensity (BSD-Q/DRAQ). Whereas the CSCS-REM produces a relative measure reflecting deviations from wakefulness, the comparison instruments aim to capture the absolute strength of experience. The partial correspondence between relative and absolute representations may therefore account for correlations that are statistically significant yet modest in magnitude.

### Limitations

This study has several limitations. First, the data collection method relied on retrospective self-report. Even if participants experienced certain phenomenological contents during dreams, they may have failed to report them due to difficulties in recall. This raises the possibility of memory distortions and recall bias, a constraint inherent to retrospective self-report given the reconstructive nature of memory retrieval (Conway and Pleydell-Pearce 2000; Conway and Loveday 2015). For this reason, administering the scale immediately after the dream is recommended, consistent with the principle of temporal proximity in dream reporting (Windt 2013); its applicability is nevertheless lost when no dream is recalled. In addition, some participants may have refrained from reporting socially sensitive dream contents due to concerns about social desirability or privacy. Furthermore, the nature of consciousness is highly dynamic, fluctuating on a scale of seconds, and the act of reporting itself may alter its contents. Although language is the primary tool for describing states of consciousness, its context dependence, ambiguity, and inherent limits prevent it from fully capturing the diversity of subjective experience (Gamez 2014). Moreover, the process of reporting may interrupt the stream of consciousness and thereby transform the very phenomenon under study (Hurlburt and Schwitzgebel 2007). This constitutes an unavoidable methodological limitation of the current approach and reflects a broader challenge in consciousness research. Second, the sample was relatively homogeneous, with a pronounced gender imbalance, as female participants substantially outnumbered males. This imbalance may partly reflect the higher dream recall frequencies consistently reported by women (Schredl et al. 1998; Schredl and Reinhard 2008; Nielsen 2012). Similar patterns of gender imbalance have been observed in dream questionnaire validation and longitudinal dream research, particularly in studies where recall is a prerequisite for participation (*e.g.*  Napias et al. 2021; Scarpelli et al. 2022; Voss et al. 2013, Sample 1). Regardless of its origins, this imbalance limits the generalizability of the findings and constrains interpretations regarding gender differences. The sample was also predominantly composed of university-educated participants, which may further restrict generalizability to populations with lower educational attainment. Future studies with more balanced gender and educational representation are needed to further examine the scale's psychometric properties across diverse demographic groups. Third, although the use of self-report methods in evaluating phenomenological contents is methodologically unavoidable, it is not sufficient on its own. Administering the scale together with neurophysiological measures could help strengthen validity evidence; however, such methods cannot directly capture the qualitative dimensions of subjective experience (qualia) (Koch et al. 2016). Qualitative analyses of dream reports also represent one of the useful approaches for uncovering phenomenological content, yet this strategy remains limited because it relies on subjective narration and open-ended interpretation. Situated between these two approaches, the CSCS-REM offers an intermediate methodological solution: it preserves access to the phenomenological dimension while providing a standardized, quantifiable structure that open-ended reports do not, by assessing phenomenological experience through relative differences referenced to waking consciousness. Memory recall remains a shared limitation of all phenomenological self-report methods.

## Conclusion

In summary, this study introduces a psychometric instrument designed to comparatively assess REM dream consciousness and waking consciousness. The resulting factor structure enabled an examination of the phenomenological diversity of dream consciousness and provided a unified basis for evaluating theoretical models of different states of consciousness within a single measurement system. The CSCS-REM also holds potential as a complementary source of data for clinical applications. Clinically, examining differences in emotional tone between REM dream experiences and waking consciousness in major depressive disorder may provide insights into mechanisms of mood regulation. In schizophrenia spectrum disorders, evaluating dream phenomenology could help to elucidate alterations in reality perception, cognitive coherence, and reflective self-awareness. In neurodegenerative conditions, analyzing the cognitive and emotional features of REM content may contribute to identifying markers of cognitive decline or impairments in emotional processing. Moreover, in post-traumatic stress disorder, anxiety disorders, and chronic pain syndromes, quantitatively assessing the phenomenological divergence between dream and waking consciousness may offer an additional dimension for monitoring treatment effectiveness. Future research should conduct measurement invariance testing across gender, age, and educational groups to establish whether the factor structure, item loadings, and intercepts remain stable across subpopulations. The psychometric properties of the scale should also be examined across other states of consciousness (*e.g.* non-REM sleep, hypnagogic and hypnopompic states, meditation, dissociative experiences), which may advance understanding of both continuity and discontinuity in consciousness phenomenology. Independent adaptation and validation studies are also an important future direction to enable the scale's use in other linguistic and cultural contexts. The English translation provided in Appendix B is intended to serve as a starting point for such work and will require cross-cultural adaptation and psychometric validation before use.

## Supplementary Material

Appendix_A-B_niag038

## Data Availability

The data from this study, along with item files and scoring instructions for the CSCS-REM, are available in the Files section of our Open Science Framework project (https://osf.io/y3pvs).

## Notes

This work was supported within the framework of the European Cooperation in Science and Technology (COST) Programme under the TÜBİTAK Grant No. 122R049. The project, titled ‘Comparing REM Dream Consciousness and Waking Consciousness: Phenomenological and Neurobiological Structure,’ was conducted as part of the Electronic and Informatics Research Support Group (EEAG).
