# Supplementary material for: Bridging dream and wake states: a new tool for consciousness research
Source: Neurosci Conscious. 2026 Jul 15;2026(1):niag038. doi: 10.1093/nc/niag038 (PMC13371965; doi:10.1093/nc/niag038)
Supplement: Appendix_A-B_niag038 [file appendix_a-b_niag038.docx]

**Appendix A. Consciousness States Comparison Scale: REM Version (CSCS-REM)**

**FARKLI BİLİNÇ DURUMLARINI KARŞILAŞTIRMA ÖLÇEĞİ: REM RÜYASI VERSİYONU (FBD-R)**

- Az önce gördüğünüz rüyadaki deneyimlerinizi uyanıklık durumunuzla kıyaslayarak maddeleri yanıtlayın.
- Doğru ya da yanlış cevap olmadığından, yanıtlarınız öznel deneyimlerinizi yansıtmalıdır. Yanıtlarınızı önceki maddelere göre değiştirmekten veya sosyal olarak istenen yanıtları sağlamaya çalışmaktan kaçının.
- Lütfen her bir maddeyi cevapladığınızdan emin olun.
- Her maddede, rüyadaki yaşantınızın uyanıklıktakinden ne ölçüde farklılaştığını yansıtan şıkkı işaretleyin.
- Okuduğunuz bir madde sizin için geçerli değilse, örneğin o ifadedeki şeyi rüyanızda deneyimlemediyseniz ‘Yanıtım yok (0)’ şıkkını işaretleyin.
- Rüyadaki yaşantınız, uyanıklık durumunuzdaki yaşantınız ile aynı ise 3’ü işaretleyin.

***Örnek****: Ağrı deneyimi*

0 puan (Yanıtım yok) = Az önce gördüğümde rüyada ağrı deneyimlemedim

1 puan = Az önce gördüğüm rüyada, uyanıklık durumuma kıyasla **ağrı deneyimim** çok düşük düzeydeydi.

2 puan = Az önce gördüğüm rüyada, uyanıklık durumuma kıyasla **ağrı deneyimim** düşük düzeydeydi.

3 puan = Az önce gördüğüm rüyada, uyanıklık durumuma kıyasla **ağrı deneyimim** aynı düzeydeydi.

4 puan = Az önce gördüğüm rüyada, uyanıklık durumuma kıyasla **ağrı deneyimim** yüksek düzeydeydi.

5 puan = Az önce gördüğüm rüyada, uyanıklık durumuma kıyasla **ağrı deneyimim** çok yüksek düzeydeydi.

| **Az önce gördüğünüz rüyayı göz önünde bulundurarak, aşağıda yer alan ifadelerden sizin için en uygun olanını uyanıklık durumunuzla kıyaslayarak işaretleyiniz.**  **Aşağıdaki maddelerde ifade edilen durumu rüyada deneyimlemediyseniz “Yanıtım yok (0)” şıkkını işaretleyiniz.** | | | | | | | |
| --- | --- | --- | --- | --- | --- | --- | --- |
| **“Az önce gördüğüm rüyada, uyanıklık durumuma kıyasla …”** | | Yanıtım yok  (0) | **Çok düşük** düzeydeydi  (1) | **Düşük** düzeydeydi  (2) | **Aynı** düzeydeydi  (3) | **Yüksek** düzeydeydi  (4) | **Çok yüksek** düzeydeydi  (5) |
| 1. | … tat deneyimim |  |  |  |  |  |  |
| 2. | … koku deneyimim |  |  |  |  |  |  |
| 3. | … denge deneyimim |  |  |  |  |  |  |
| 4. | … dokunma deneyimim |  |  |  |  |  |  |
| 5. | … işitme deneyimim |  |  |  |  |  |  |
| 6. | … görme deneyimim |  |  |  |  |  |  |
| 7. | … açlık, susuzluk gibi bedensel duyumlarımı fark edebilmem |  |  |  |  |  |  |
| 8. | … olaylara farklı açılardan bakıp, değerlendirebilmem |  |  |  |  |  |  |
| 9. | … kararlarıma etki eden düşüncelerimi fark edebilmem |  |  |  |  |  |  |
| 10. | … kararlarıma etki eden duygularımı fark edebilmem |  |  |  |  |  |  |
| 11. | … olayların sebeplerini kavrayabilmem |  |  |  |  |  |  |
| 12. | … olayların sonuçlarını öngörebilmem |  |  |  |  |  |  |
| 13. | … geleceğe dair planlar yapabilmem |  |  |  |  |  |  |
| 14. | … düşüncelerim üzerindeki kontrolüm |  |  |  |  |  |  |
| 15. | … düşüncelerim ve duygularım üzerine düşünebilmem |  |  |  |  |  |  |
| 16. | … başkalarının ne düşündüğünü anlayabilmem |  |  |  |  |  |  |
| 17. | … mantıklı düşünebilme ve muhakeme becerim |  |  |  |  |  |  |
| 18. | … dili akıcı bir şekilde kullanabilmem |  |  |  |  |  |  |
| 19. | … çevremdekilere odaklanabilmem |  |  |  |  |  |  |
| 20. | … zihinsel süreçlerime odaklanabilmem |  |  |  |  |  |  |
| 21. | … dikkatimi sürdürebilmem |  |  |  |  |  |  |
| 22. | … dikkatimi gerektiğinde yönlendirebilmem |  |  |  |  |  |  |
| 23. | … yaşadığım olayların ayrıntılarını hatırlayabilmem |  |  |  |  |  |  |
| 24. | … tarihsel olay ve olguları hatırlayabilmem |  |  |  |  |  |  |
| 25. | … öğrendiğim yeni bilgileri hatırlayabilmem |  |  |  |  |  |  |
| 26. | … algıladığım zaman ile fiziksel dünyada akan zaman arasındaki uyum |  |  |  |  |  |  |
| 27. | … geçmiş, şimdi ve gelecek arasındaki zamansal devamlılığı anlayabilmem |  |  |  |  |  |  |
| 28. | … geçen zamanı saate bakmaksızın doğru şekilde tahmin edebilmem |  |  |  |  |  |  |
| 29. | … saatin kaç olduğuna dair farkındalığım |  |  |  |  |  |  |
| 30. | … nerede olduğuma dair farkındalığım |  |  |  |  |  |  |
| 31. | … etrafımdaki yerlerin konumunu doğru şekilde tarif edebilme kabiliyetim |  |  |  |  |  |  |
| 32. | … yerler arasındaki mesafeleri doğru şekilde tahmin edebilme kabiliyetim |  |  |  |  |  |  |
| 33. | … deneyimlediğim duyguların yoğunluğu |  |  |  |  |  |  |
| 34. | … olumsuz duygu deneyimim |  |  |  |  |  |  |
| 35. | … duygularımı adlandırabilmem |  |  |  |  |  |  |

**Puanlama**

Farklı Bilinç Durumlarını Karşılaştırma Ölçeği: REM Rüyası Versiyonu (FBD-R), sekiz alt boyut ve toplam ölçek puanı üzerinden değerlendirilmektedir. Alt boyut puanları, ilgili maddelerin toplamı ya da ortalaması alınarak hesaplanabilir. Toplam kullanıldığında ölçekten alınabilecek en düşük toplam puan 0, en yüksek toplam puan ise 175'tir. Alt boyutlar için hesaplanan puan aralıkları madde sayılarına göre değişmektedir: Üstbilişsel Farkındalık alt boyutunda 0-50, Bilişsel Akıcılık alt boyutunda 0-25, Mekânsal Farkındalık alt boyutunda 0-15, Zamansal Farkındalık alt boyutunda 0-20, Duygusal Farkındalık alt boyutunda 0-15, Bellek alt boyutunda 0-15, İçsel ve Kimyasal Duyular alt boyutunda 0-15 ve Algısal ve Bedensel Duyular alt boyutunda 0-20 arasında puan elde edilebilmektedir. Ortalama puan hesaplandığında ise tüm alt boyutlar 0-5 aralığında karşılaştırılabilir hale gelmektedir.

Alt boyutlara ait madde numaraları aşağıda verilmiştir:

- İçsel ve Kimyasal Duyular (ICS): 1, 2, 7
- Algısal ve Bedensel Duyular (PBS): 3, 4, 5, 6
- Üstbilişsel Farkındalık (MA): 8, 9, 10, 11, 12, 13, 14, 15, 16, 17
- Bilişsel Akıcılık (CF): 18, 19, 20, 21, 22
- Bellek (MEM): 23, 24, 25
- Zamansal Farkındalık (TA): 26, 27, 28, 29
- Mekânsal Farkındalık (SA): 30, 31, 32
- Duygusal Farkındalık (EA): 33, 34, 35

Ortalama puanın 3’e yakın olması rüya deneyiminin uyanıklıkla benzer olduğunu; 3’ten yüksek değerler daha yoğun, 3’ten düşük değerler ise daha düşük düzeyde deneyimi göstermektedir. “0” yanıtı, ilgili deneyimin rüyada yaşanmadığını veya yanıt verilmediğini ifade eder; bu değerler ayrıca var–yok biçiminde ayrı bir değişken olarak da işlenebilir. Ölçek ters madde içermemektedir.

**Appendix B. Consciousness States Comparison Scale: REM Dream Version (CSCS-REM)**

- Please respond to the items by comparing your experiences in the most recent dream you recalled with your experiences during wakefulness.
- There are no right or wrong answers; your responses should reflect your subjective experience. Avoid changing your responses based on previous items or attempting to provide socially desirable answers.
- Please make sure to answer every item.
- For each item, indicate the option that best reflects how different your dream experience was compared to your waking experience.
- If an item does not apply to you (e.g., you did not experience that phenomenon in your dream), select “No response (0).”
- If your dream experience was the same as your waking experience, select “3.”

***Example:*** *Pain Experience*

0 points (No response) = In the dream I just recalled, I did not experience any pain.

1 point = In the dream I just recalled, compared to when I am awake, my experience of pain was very low.

2 points = In the dream I just recalled, compared to when I am awake, my experience of pain was low.

3 points = In the dream I just recalled, compared to when I am awake, my experience of pain was the same.

4 points = In the dream I just recalled, compared to when I am awake, my experience of pain was high.

5 points = In the dream I just recalled, compared to when I am awake, my experience of pain was very high.

| **Considering the most recent dream you recalled, please respond to the following items by comparing your experience in the dream with your experience during wakefulness.**  **If you did not experience the phenomenon described in an item during the dream, please select “No response (0).”** | | | | | | | |
| --- | --- | --- | --- | --- | --- | --- | --- |
| **“In the dream I just recalled, compared to when I am awake, …”** | | No response / Did not experience it (0) | **Very low**  (1) | **Low**  (2) | **The same**  (3) | **High**  (4) | **Very high**  (5) |
| 1. | … my taste experience |  |  |  |  |  |  |
| 2. | … my smell experience |  |  |  |  |  |  |
| 3. | … my balance experience |  |  |  |  |  |  |
| 4. | … my touch experience |  |  |  |  |  |  |
| 5. | … my hearing experience |  |  |  |  |  |  |
| 6. | … my vision experience |  |  |  |  |  |  |
| 7. | … noticing bodily sensations such as hunger or thirst |  |  |  |  |  |  |
| 8. | … viewing and evaluating events from different perspectives |  |  |  |  |  |  |
| 9. | … noticing the thoughts that affect my decisions |  |  |  |  |  |  |
| 10. | … noticing the feelings that affect my decisions |  |  |  |  |  |  |
| 11. | … understanding the causes of events |  |  |  |  |  |  |
| 12. | … anticipating the outcomes of events |  |  |  |  |  |  |
| 13. | … making plans for the future |  |  |  |  |  |  |
| 14. | … my control over my thoughts |  |  |  |  |  |  |
| 15. | … reflecting on my thoughts and feelings |  |  |  |  |  |  |
| 16. | … understanding what others are thinking |  |  |  |  |  |  |
| 17. | … my ability to think logically and reason |  |  |  |  |  |  |
| 18. | … my ability to use language fluently |  |  |  |  |  |  |
| 19. | … my ability to focus on my surroundings |  |  |  |  |  |  |
| 20. | … my ability to focus on my mental processes |  |  |  |  |  |  |
| 21. | … my ability to sustain attention |  |  |  |  |  |  |
| 22. | … my ability to direct my attention when needed |  |  |  |  |  |  |
| 23. | … my ability to remember details of events I have experienced |  |  |  |  |  |  |
| 24. | … my ability to remember historical events and facts |  |  |  |  |  |  |
| 25. | … my ability to remember newly learned information |  |  |  |  |  |  |
| 26. | … the match between the time I perceive and the actual passage of time |  |  |  |  |  |  |
| 27. | … my ability to understand the temporal continuity between past, present, and future |  |  |  |  |  |  |
| 28. | … my ability to estimate the passage of time correctly without looking at a clock |  |  |  |  |  |  |
| 29. | … my awareness of what time it is |  |  |  |  |  |  |
| 30. | … my awareness of where I am |  |  |  |  |  |  |
| 31. | … my ability to describe the location of places around me accurately |  |  |  |  |  |  |
| 32. | … my ability to estimate the distances between places accurately |  |  |  |  |  |  |
| 33. | … the intensity of the emotions I experience |  |  |  |  |  |  |
| 34. | … my experience of negative emotions |  |  |  |  |  |  |
| 35. | … my ability to label my emotions |  |  |  |  |  |  |

*Note.* The validity and reliability analyses of the Consciousness States Comparison Scale: REM Version (CSCS-REM) were conducted with a Turkish-speaking sample. The English version of the scale included in the appendix is a translation prepared independently by two experts; however, it has not yet been psychometrically validated in an English-speaking sample. Therefore, while the Turkish version of the scale has established psychometric properties, the English version should be considered a preliminary translation and requires further testing before being used in research.

**Scoring**

The Consciousness States Comparison Scale: REM Version (CSCS-REM) is evaluated through eight subscales as well as a total scale score. Subscale scores can be calculated either by summing or averaging the scores of the relevant items. When sum scores are used, the minimum possible total score is 0 and the maximum possible total score is 175. The score ranges for the subscales vary according to the number of items: Metacognitive Awareness 0–50, Cognitive Fluency 0–25, Spatial Awareness 0–15, Temporal Awareness 0–20, Emotional Awareness 0–15, Memory 0–15, Interoceptive and Chemical Senses 0–15, and Perceptual and Bodily Senses 0–20. When mean scores are calculated, all subscales can be directly compared on a common 0–5 scale.

The item numbers belonging to each subscale are as follows:

- Interoceptive and Chemical Senses (ICS): 1, 2, 7
- Perceptual and Bodily Senses (PBS): 3, 4, 5, 6
- Metacognitive Awareness (MA): 8, 9, 10, 11, 12, 13, 14, 15, 16, 17
- Cognitive Fluency (CF): 18, 19, 20, 21, 22
- Memory (MEM): 23, 24, 25
- Temporal Awareness (TA): 26, 27, 28, 29
- Spatial Awareness (SA): 30, 31, 32
- Emotional Awareness (EA): 33, 34, 35

A mean score close to 3 indicates that the dream experience was similar to the waking experience; values above 3 indicate a more intense experience, while values below 3 indicate a less intense experience compared to wakefulness. A response of 0 means that the participant did not have that particular experience in the dream or chose not to respond; such responses can also be treated as a separate variable in the form of a presence–absence indicator. The scale does not include any reverse-coded items.
